# nNOS-Expressing Neurons in the Ventral Tegmental Area and Substantia Nigra Pars Compacta

**DOI:** 10.1523/ENEURO.0381-18.2018

**Published:** 2018-11-16

**Authors:** Eleanor J. Paul, Eliza Kalk, Kyoko Tossell, Elaine E. Irvine, Nicholas P. Franks, William Wisden, Dominic J. Withers, James Leiper, Mark A. Ungless

**Affiliations:** 1 MRC London Institute of Medical Sciences (LMS), London W12 0NN, United Kingdom; 2 Institute of Clinical Sciences (ICS), Faculty of Medicine, Imperial College London, London W12 0NN, United Kingdom; 3 Department of Life Sciences, Imperial College London, South Kensington, London SW7 2AZ, United Kingdom

**Keywords:** GABA, glutamate, nNOS, SNc, VTA

## Abstract

GABA neurons in the VTA and SNc play key roles in reward and aversion through their local inhibitory control of dopamine neuron activity and through long-range projections to several target regions including the nucleus accumbens. It is not clear whether some of these GABA neurons are dedicated local interneurons or if they all collateralize and send projections externally as well as making local synaptic connections. Testing between these possibilities has been challenging in the absence of interneuron-specific molecular markers. We hypothesized that one potential candidate might be neuronal nitric oxide synthase (nNOS), a common interneuronal marker in other brain regions. To test this, we used a combination of immunolabelling (including antibodies for nNOS that we validated in tissue from nNOS-deficient mice) and cell type-specific virus-based anterograde tracing in mice. We found that nNOS-expressing neurons, in the parabrachial pigmented (PBP) part of the VTA and the SNc were GABAergic and did not make detectable projections, suggesting they may be interneurons. In contrast, nNOS-expressing neurons in the rostral linear nucleus (RLi) were mostly glutamatergic and projected to a number of regions, including the lateral hypothalamus (LH), the ventral pallidum (VP), and the median raphe (MnR) nucleus. Taken together, these findings indicate that nNOS is expressed by neurochemically- and anatomically-distinct neuronal sub-groups in a sub-region-specific manner in the VTA and SNc.

## Significance Statement

GABA neurons in the VTA and SNc play important roles in reward and aversion through their local control of dopamine neuron activity and long-range projections to regions such as the nucleus accumbens. It is not clear whether some of these neurons are dedicated interneurons, or if they all project externally and synapse locally. We find that neuronal nitric oxide synthase (nNOS) is expressed by some GABAergic neurons that do not make detectable projections, suggesting that they may be interneurons. In addition, nNOS is expressed by a subgroup of glutamatergic neurons that project to regions including the ventral pallidum (VP) and median raphe (MnR) nucleus. Our study paves the way for future investigation of the function of these molecularly-defined populations.

## Introduction

Around one third of neurons in the VTA and SNc are GABAergic (Olson and Nestler, 2007; Nair-Roberts et al., 2008). These neurons make local, inhibitory synaptic connections with dopamine neurons and their activation can drive conditioned place aversion and reduce food consumption (Omelchenko and Sesack, 2009; Tan et al., 2012; van Zessen et al., 2012). In addition, they send long-range axonal projections to several target regions, including the nucleus accumbens where they can regulate associative learning (Brown et al., 2012; Taylor et al., 2014). It is not clear whether a subset of these GABA neurons are dedicated local interneurons or if they all collateralize and send projections externally as well as making local synaptic connections. Testing between these possibilities has been challenging in the absence of interneuron-specific molecular markers. Indeed, of the cardinal interneuron markers used to identify and selectively target sub-populations of interneurons in other regions of the brain, most are either not expressed in either the VTA or SNc, or are also expressed by sub-groups of dopamine neurons (e.g., somatostatin, cholecystokinin, vasoactive intestinal peptide, neuropeptide Y, parvalbumin, and calretinin; Hökfelt et al., 1980; Seroogy et al., 1988, 1989; Rogers, 1992; Isaacs and Jacobowitz, 1994; Liang et al., 1996; Gonzalez-Hernandez and Rodriguez, 2000; Klink et al., 2001; Lein et al., 2007; Olson and Nestler, 2007; Dougalis et al., 2012; Merrill et al., 2015). One potential candidate, however, is neuronal nitric oxide synthase (nNOS). nNOS is a member of the NOS family of enzymes that catalyze the synthesis of NO from L-arginine (Knowles et al., 1989; Garthwaite, 1991). In the nervous system NO acts as a gaseous transmitter that can move rapidly across plasma membranes in anterograde and retrograde directions (Garthwaite and Boulton, 1995; Wang and Marsden, 1995). In several brain regions nNOS is selectively expressed by specific types of GABAergic interneurons (Klausberger and Somogyi, 2008; Tepper et al., 2010). Although several reports indicate that nNOS is expressed sparsely in the VTA and/or the SNc, there are discrepancies regarding the extent of its expression, which sub-regions it is expressed in, and the degree of colocalization with tyrosine hydroxylase (TH; the rate limiting enzyme in dopamine synthesis that is most commonly used to identify dopamine neurons; Vincent and Kimura, 1992; Rodrigo et al., 1994; Gonzalez-Hernandez and Rodriguez, 2000; Backes and Hemby, 2003; Klejbor et al., 2004; Gotti et al., 2005; Cavalcanti-Kwiatkoski et al., 2010; Mitkovski et al., 2012). We hypothesized that some of these discrepant findings may have arisen because of non-specific immunolabelling. To address this directly, we tested three different nNOS antibodies for reliable immunolabelling in the VTA and SNc, using tissue from nNOS-deficient mice as a control. This allowed us to establish that only one of these antibodies exhibited reliable immunolabelling in the VTA and SNc. Using this antibody, combined with cell type-specific viral-based anterograde axonal tracing, we found that nNOS is expressed by several distinct sub-groups of neurons in the VTA and SNc, including GABAergic neurons that do not appear to make projections and may therefore be interneurons, and glutamatergic projection neurons.

## Materials and Methods

### Animal maintenance and breeding

C57Bl/6NCrl (RRID: IMSR_CRL:27; WT) mice were purchased from Charles River. nNOS-deficient (RRID: IMSR_JAX:002986), NOS1Cre (RRID: IMSR_JAX:017526), VGATCre (vesicular GABA transporter; RRID: IMSR_JAX:016962), and RiboTag (RRID: IMSR_JAX:011029) mice were purchased from The Jackson Laboratory. Mice heterozygous for VGATCre (VGATCre -/+) were crossed with mice homozygous for RPL22^HA^ (RiboTag +/+) producing VGATCre -/+ RiboTag -/+ offspring (VGATCre:RiboTag). NOS1Cre mice were heterozygous. All breeding and experimental procedures were conducted in accordance with the Animals (Scientific Procedures) Act of 1986 (United Kingdom) and approved by Imperial College London’s Animal Welfare and Ethical Review Body. All mice were maintained in social groups of two to four, where possible, with appropriate environmental enrichment (e.g., bedding and tunnels). They were kept in rooms at a constant temperature and maintained on a 12/12 h light/dark cycle. They were fed on standard rodent chow and water *ad libitum*.

### Tissue fixation and preparation

C57Bl/6NCrl, nNOS-deficient, VGATCre:RiboTag, or NOS1Cre mice were anaesthetized under isoflurane (4%) and given a lethal intraperitoneal injection of pentobarbital (100 mg/ml; Euthatal). They were transcardiallly perfused with 50 ml of ice-cold PBS followed by 50–100 ml of 4% paraformaldehyde (PFA; Sigma Aldrich) in PBS. When fixed, the brains were removed and placed in 10 ml of 4% PFA for 1 h post-fixation at room temperature. After three washes in PBS, brains were placed in 30% sucrose (Sigma Aldrich) dissolved in PBS for cryo-protection, and kept at 4°C for 24–48 h. Subsequently, all brains were embedded in optimal cutting temperature (OCT) medium and snap frozen in isopentane (2-methlybutane) at -55°C. All tissue was then stored at -80°C until sectioning.

### Immunocytochemistry

All immunolabelling was conducted on tissue from mice aged 8–12 weeks old. Brains were sectioned using a Leica CM1800 cryostat (Leica Microsystems). Coronal sections (30 µm) were taken from the midbrain, or from the whole brain in the case of Nos1Cre mice. Free floating sections were washed in PBS for 10 min at room temperature. Following this, they were blocked in 6% normal donkey serum (NDS) in 0.2% Triton X-100 in PBS (PBSTx) for 60 min at room temperature. Primary antibodies (Table 1) were diluted in 2% donkey serum in PBSTx, and sections were incubated in the primary antibody solutions overnight at 4°C. Sections were washed (3 × 10 min) in PBS at room temperature. Secondary antibodies (Table 2) were diluted in 2% donkey serum 0.2% PBSTx. Sections were incubated in secondary antibody solution for a minimum of 1.5 h at room temperature. They were then washed (3 × 10 min) in PBS. Stained sections were mounted onto glass microscope slides and when dry were cover-slipped using VectaShield mounting medium (Vector Laboratories). SNc and VTA regions were determined using tyrosine hydroxylase (TH) expression. Region outlines were traced from Franklin and Paxinos (2008).

**Table 1.** Primary antibodies

Antibody	Host species	Supplier (catalog number; RRID)	Concentration
Anti-TH	Chicken	Abcam (ab76442; AB_1524535)	1:1000
Anti-nNOS	Mouse	Sigma Aldrich (N2280; AB_260754)	1:500
Anti-nNOS	Rabbit	Cell Signalling (4234; AB_10694499)	1:500
Anti-nNOS	Rabbit	Sigma Aldrich (N7155; AB_260795)	1:500
Anti-HA	Mouse	Sigma Aldrich (H3663; AB_262051)	1:1000
Anti-HA	Rabbit	Abcam (ab9110; AB_307019)	1:500
Anti-5HT	Rabbit	ImmunoStar (20080; AB_572263)	1:2000
Anti-VGLUT2	Rabbit	Alomone (AGC036; AB_2340950)	1:500
Anti-VGAT	Rabbit	SYSY (131 003; AB_887869)	1:500
Anti-substance P	Guinea pig	Abcam (ab10353; AB_297089)	1:500
Anti-AADC	Rabbit	Millipore (AB1569; RRID:AB_90789)	1:500
Anti-DAT	Rat	Millipore (MAB369; RRID:AB_2190413)	1:500

**Table 2.** Secondary antibodies

Antibody	Conjugation	Host species	Supplier (catalog number; RRID)	Concentration
Anti-chicken	Alexa Fluor 488	Goat	Thermo Fisher Scientific (A-11039; AB_2534096)	1:1000
Anti-chicken	Cy3	Donkey	Jackson ImmunoResearch Labs (703-165-155; AB_2340363)	1:1000
Anti-mouse	Cy3	Donkey	Jackson ImmunoResearch Labs (715-165-150: AB_2340813)	1:1000
Anti-mouse	Cy5	Donkey	Jackson ImmunoResearch Labs (715-175-151; AB_2340820)	1:1000
Anti-rabbit	Alexa Fluor 633	Goat	Thermo Fisher Scientific (A21070; AB_2535731)	1:1000
Anti-rabbit	Cy3	Donkey	Jackson ImmunoResearch Labs (711-165-152; AB_2307443)	1:1000
Anti-goat	Alexa Fluor 488	Donkey	Thermo Fisher Scientific (A11055; AB_2534102)	1:1000
Anti-guinea pig	Alexa Fluor 488	Goat	Thermo Fisher Scientific (A11073: AB_2534117)	1:1000
Anti-rat	Alexa Fluor 488	Goat	Thermo Fisher Scientific (A-11006; RRID:AB_2534074)	1:1000

### Microscopy

Confocal images were acquired using a Leica SP5 confocal microscope with the pinhole set at 1 Airy unit. All images were processed with Fiji software. Images of cell bodies were acquired with z-stacks of 1 μm. To determine colocalization, channels were viewed both individually and in composite. Colocalization was determined if the cell body was visible in multiple channels through its entire thickness (multiple *z*-planes). Representative examples of stacked images are shown. Images of axon terminals in nNOS+ neuron target areas were acquired with z-stacks of 0.5 μm. Ten *z*-planes were stacked and brightness, and contrast was adjusted equally across all axonal projection images for comparison. Images of synaptic terminals were acquired with z-stacks of 0.25 μm. ChR2-mCherry+ synaptic boutons were located in single *z*-planes, which were extracted from the stack to determine colocalization with VGAT or VGluT2.

### Stereotaxic injections of adeno-associated virus (AAV)

The 1-Ef1a-DIO-ChR2-mCherry construct (gifted by the Deisseroth Lab) was commercially packaged in AAV serotype 2/1 vector consisting of the AAV2 ITR genomes and the AAV1 serotype capsid gene (Vector Biolab, Philadelphia). The virus was diluted in sterile PBS and 5% glycerol (pH 7.2) to a concentration of 2.7 × 10^13^ GC/ml. All viral tracing experiments were conducted on adult (11–13 weeks) NOS1Cre (-/+) mice. Mice were briefly anaesthetized in an induction chamber with isoflurane (4%) and placed in a stereotaxic frame (David Kopf Instruments) with continued isoflurane administration (2%). The eyes were protected with Lacri-lube, the scalp was shaved, and the skin disinfected with chlorheximide. All mice received a subcutaneous injection of carprofen (Rimadyl; 5 mg/kg) for post-operative anesthesia. An incision (<1 cm) was made along the midline, and bupivacaine (2.5 mg/ml) was delivered directly to the incision site for local analgesia. A small hole was drilled in the scalp based on coordinates from bregma. Using a 33-gauge metal needle and a Hamilton syringe the virus solution (0.1 µl) was injected unilaterally at a flow rate of 0.3 µl/min. We systematically varied the injection coordinates [anterior-posterior (AP) -3.0–3.4 mm, medial-lateral (ML) 0.4–0.9 mm, dorsal-ventral (DV) 4.3–4.8 mm] to obtain labeling of different sub-regions. The flow rate was controlled by a programmable pump (Elite Nanomite Infusion/Withdrawal Programmable Pump 11, 704507, Harvard Apparatus). After injection, the needle was left in place for 5 min to allow for the spread of the virus. The incision was then sutured using nylon monofilament, non-absorbable sutures (size 2-0, 95060-062, VWR). Mice were allowed to recover in a heated chamber (30°C) before being placed back into their home cage with littermates. All mice were monitored for five days after surgery, during which time they had access to carprofen (Rimadyl; 50 mg/ml) in their drinking water. Two weeks after surgery, the mice underwent transcardial perfusion, as described above, and tissue processed for microscopy.pAAV-hSyn-DIO-mCherry was a gift from Bryan Roth (Addgene plasmid #50459). The pAAV transgene plasmid was packaged into a mixture of serotypes AAV1 and AAV2 (1:1) as previously described (Klugmann et al., 2005; Yu et al., 2015). All other details were the same as for the DIO-ChR2-mCherry experiments, except that the AAV was injected using a Nanoject III Programmable Nanolitre Injector (Drummond Scientific; 3-000-207) with a mineral oil filled glass micropipette. A volume or either 10 or 30 nl was injected at a rate of 3 nl/s, and then the needle was left in position for 10 min to allow for spread of the virus.

### Experimental design and statistics

#### Wild type versus nNOS deficient

To compare nNOS antibody staining in wild-type and nNOS-deficient mice, the experimenter was blind to the strain of the mouse from the stage of immunolabelling until after image analysis. Mice for each experimental group were stained in parallel to control for differences between staining experiments. All images in this section were obtained with matched confocal settings. Each anti-nNOS antibody was tested in a total of three male WT and three male nNOS-deficient mice. The concentration of nNOS antibody was optimized through staining and imaging at three concentrations (1:250, 1:500, 1:1000). Images from the optimum concentration of 1:500 are shown.

#### Quantification of nNOS-expressing neurons

For the quantification of nNOS-expressing neurons triple immunolabelling for nNOS, HA, and TH was conducted in three male VGATCre:RiboTag mice. To obtain estimates of the numbers of nNOS neurons, and their neurotransmitter phenotype, every fourth midbrain section was selected for staining and imaging. Tile-scans were taken of the entire VTA and SNc visible on the right-hand side of the brain section. Merged tile-scan images were processed using Fiji (ImageJ) and VTA and SNc sub-region anatomy was defined based on TH expression. HA+ cells, nNOS+ cells and HA+/nNOS+ were counted in each sub-region using the ImageJ cell counter plugin.

#### nNOS neuron circuit tracing and ChR2-mCherry colocalization

A total of 18 (eight males and 10 females) virus injected NOS1Cre mice exhibited ChR2-mCherry expression in the VTA and SNc. Eight of these mice also exhibited ChR2-mCherry expression in the supramamillary nucleus and were therefore excluded from further investigation. The remaining 10 mice were used to examine the axonal projections of nNOS+ neurons and further immunolabelling experiments. To investigate the colocalization of ChR2-mCherry, nNOS and TH, images of sub-regions were processed using Fiji (ImageJ). All ChR2-mCherry+ cells were counted in each image using the ImageJ cell counter plugin.

#### Statistics

Data are presented as mean ± SEM. Statistical comparisons were made using one-way ANOVA and Newman–Keuls *post hoc* tests, where appropriate (Prism, GraphPad Software Inc).

## Results

### Comparison of three different anti-nNOS antibodies in the midbrain of wild-type and nNOS-deficient mice

**Figure 1.**
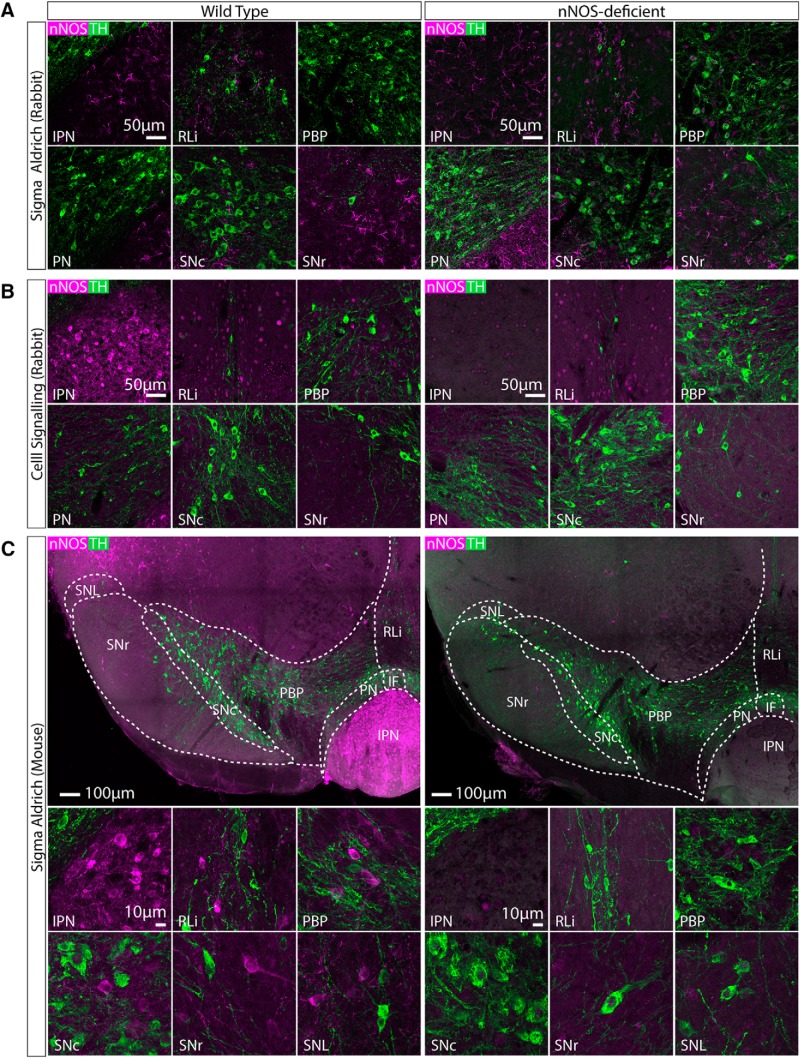
Comparison of three different anti-nNOS antibodies (for details, see Tables 1, 2) in the midbrain of wild-type and nNOS-deficient mice. Representative images of double immunolabelling for nNOS (magenta) and TH (green). ***A***, Anti-nNOS (Sigma Aldrich; N7155; AB_260795) exhibited non-specific immunolabelling that was also seen in tissue from nNOS-deficient mice. ***B***, Anti-nNOS (Cell Signaling; 4234; AB_10694499) also exhibited somewhat non-specific immunolabelling, that was only partially absent in tissue from nNOS-deficient mice. ***C***, Anti-nNOS (Sigma Aldrich; N2280; AB_260754) exhibited specific immunolabelling that was absent in tissue from nNOS-deficient mice. In wild-type tissue, nNOS+ cells were observed in the IPN, RLi, PBP, SNc, SNr, and substantia nigra pars lateralis (SNL). There was no colocalization between nNOS and TH.

We first wanted to identify a reliable nNOS antibody for use in the VTA and SNc. We tested three different commercially available antibodies (Tables 1, 2). We initially tested each antibody at three different concentrations (1:1000, 1:500, 1:250). For all three antibodies the 1:500 concentration appeared optimal in terms of reliably exhibiting immunolabelling in the interpeduncular nucleus (IPN) and in regions of the VTA and SNc in wild-type mice (Fig. 1). to thoroughly verify their specificity, each antibody (1:500) was used on midbrain sections from both wild-type mice (*n* = 3) and nNOS-deficient mice (*n* = 3; Huang et al., 1993) as a negative control. It is well established that there is a large population of nNOS-expressing neurons in the IPN, which lies just ventral to the VTA and was therefore well suited to act as a positive control (Vincent and Kimura, 1992; Rodrigo et al., 1994; Ascoli et al., 2008). The first antibody (Sigma Aldrich; N7155; AB_260795) failed to detect cell bodies and instead many processes were visible (Fig. 1*A*), which were also present in the nNOS-deficient tissue, suggesting that it was non-specific. The second antibody (Cell Signaling; 4234; AB_10694499) displayed some sparse immunoreactivity “spots” that could be mistaken for cell bodies within the VTA and SNc (Fig. 1*B*), which were also present in the nNOS-deficient tissue, suggesting that they were non-specific. The third antibody (Sigma Aldrich; N2280; AB_260754) exhibited clear immunolabelling of cell bodies in the wild-type tissue, which was completely absent in the nNOS-deficient tissue (Fig. 1*C*). In the wild-type tissue nNOS+ neurons were mosaically distributed throughout the SNc, and most notably in the parabrachial pigmented nucleus (PBP) and rostral linear nucleus (RLi) of the VTA. These were in close proximity to TH+ neurons, but there was no colocalization between nNOS and TH (Fig. 1*C*).

### nNOS is mostly expressed in GABAergic, non-dopaminergic neurons in the PBP part of the VTA and the SNc, and mostly in non-GABAergic, non-dopaminergic (putatively glutamatergic) neurons in the VTAR and RLi

**Figure 2.**
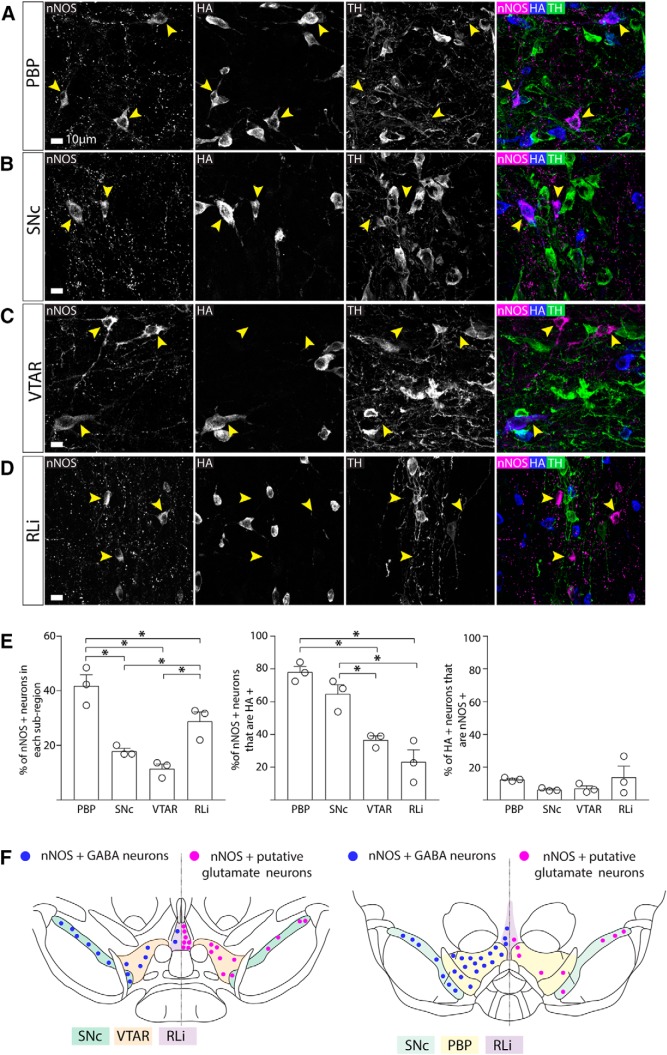
nNOS is mostly expressed in GABAergic, non-dopaminergic neurons in the PBP part of the VTA and SNc, and mostly in non-GABAergic, non-dopaminergic (putatively glutamatergic) neurons in the VTAR and RLi. ***A–D***, Representative images of triple immunolabelling for nNOS, HA, and TH, in the SNc and sub-regions of the VTA. Yellow arrows indicate nNOS+ neurons. ***E***, Graphs show the mean (±SEM; *n* = 3 mice, 1469 cells) and individual data points for percentage of nNOS+ cells localized in each region, percentage of nNOS+ cells that colocalized with HA, and the percentage of HA+ cells that were nNOS+. ***F***, Schematic illustrating the localization of nNOS+/GABAergic neurons and nNOS+/glutamatergic neurons in the VTA and SNc. Yellow arrows indicate exemplar neurons; *p < 0.05.

We next asked whether these nNOS+ neurons in the VTA and SNc were GABAergic. In the VTA and SNc, antibodies for markers of GABAergic identity (i.e., GABA, GAD, and VGAT) do not robustly label cell bodies. We, therefore, used VGATCre mice (Vong et al., 2011), where cre-recombinase is under the control of the promoter for VGAT, crossed with RiboTag mice (Sanz et al., 2009) which contains a floxed hemagglutinin (HA)-tagged exon in the RLp22 gene. The resulting offspring (VGATCre:RiboTag) exhibit robust HA expression in cell bodies in the VTA and SNc which is well suited to examining colocalization using immunolabelling (somewhat more so than standard GFP and tdTomato reporter lines, in our hands). Triple immunolabelling for nNOS, HA, and TH was conducted in midbrain sections from VGATCre:RiboTag mice (*n* = 3 mice, 1420 neurons). Nuclei sub-regions were defined using TH immunolabelling and images from a mouse brain atlas (Franklin and Paxinos, 2008). All nNOS+ and HA+ neurons within each sub-region were counted. The number of nNOS+ neurons varied in different sub-regions with the largest populations lying in the PBP of the VTA and the RLi, with smaller populations found in the SNc and VTAR (ANOVA: *F*_(3,8)_ = 22.33, *p* = 0.0003; Fig. 2*A–E*). nNOS+ neurons were almost entirely absent in the interfascicular nucleus (IF) and the paranigral nucleus (PN) and therefore these sub-regions were not included in our analysis or further investigated.

Consistent with our first set of results, there was no colocalization between TH and nNOS. In contrast, colocalization between nNOS and HA was extensive, although it varied between different sub-regions (ANOVA: *F*_(3,8)_ = 24.54, *p* = 0.0002). In the PBP and SNc, the majority nNOS+ neurons were also HA+, suggesting that nNOS+ neurons in these regions are mostly GABAergic (Fig. 2*A*,*B*,*E*,*F*). In contrast, in more rostral sub-regions (i.e., the VTAR and RLi) the majority of nNOS+ neurons were HA- (and TH-) and therefore putatively glutamatergic (Fig. 2*C–F*). Finally, the total proportion of HA+ neurons that expressed nNOS was similar in each sub-region (ANOVA: *F*_(3,8)_ = 1.268, *p* = 0.3489; Fig. 2*E*), typically <20%, indicating that nNOS+ neurons represent a sub-group of the overall GABAergic population in each of these sub-regions.

### AAV injection into the VTA and SNc of NOS1Cre± mice leads to expression of ChR2-mCherry in cell bodies in distinct regions depending on injection volume/position

**Figure 3.**
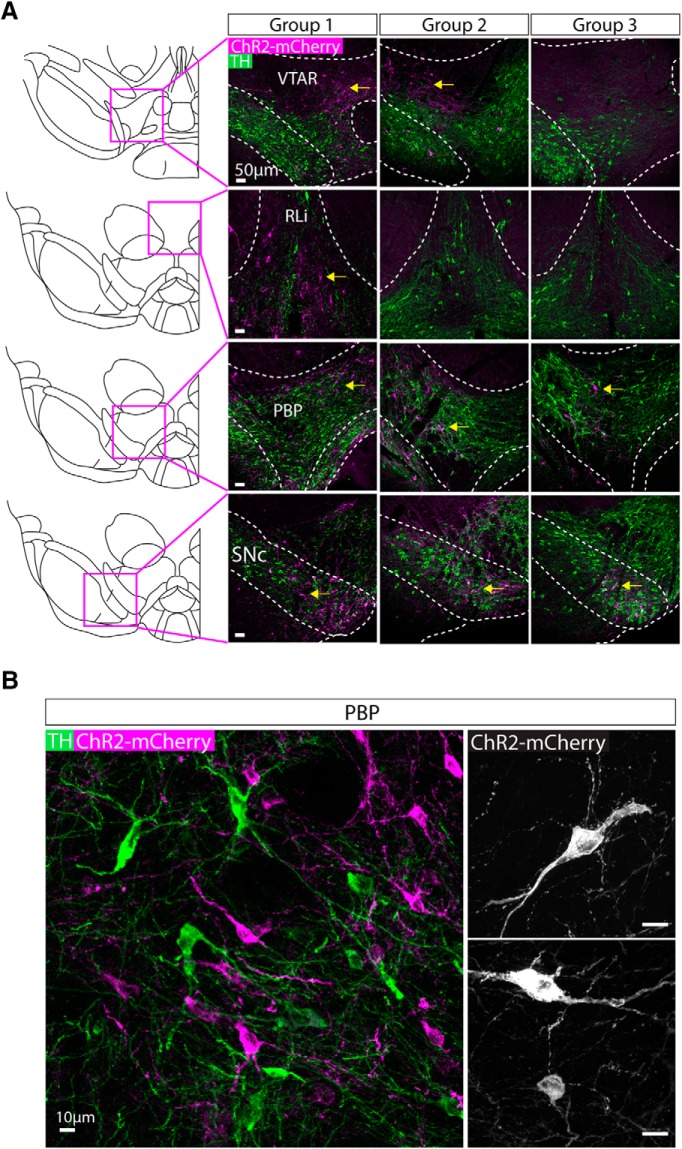
AAV injection into the VTA and SNc of NOS1cre± mice lead to expression of ChR2-mCherry in cell bodies in distinct regions depending on injection volume/position. ***A***, Representative images of ChR2-mCherry (magenta) and TH (green) in cell bodies for each injected group. Mice were grouped based on the distribution of ChR2-mCherry expressing cell bodies (yellow arrows indicate sub-regions where robust cell body expression was observed). Group 1 exhibited expression in the VTAR, RLi, PBP and SNc, group 2 exhibited expression in the dorso-lateral VTAR, PBP, and SNc, and group 3 exhibited expression that was restricted to the PBP and SNc (Table 3). ***B***, Higher magnification, representative images illustrate the robust expression of ChR2-mCherry (magenta) in cell bodies and dendrites intermingled with TH (green)-expressing neurons. Right-hand images show higher magnification images of ChR2-mCherry-expressing neurons.

Having examined the neurochemical identity of nNOS+ neurons in the VTA and SNc, we next investigated their axonal projections. To do this, we did stereotaxic injections of AAV1-Ef1a-DIO-ChR2-mCherry into the midbrain of NOS1Cre± mice (*n* = 18). We have used this AAV previously in the midbrain and hypothalamus to obtain robust ChR2-mCherry expression with no apparent consequences for cell health (Viskaitis et al., 2017; Sandhu et al., 2018). We systematically varied the injection coordinates (see Materials and Methods) and then examined the degree of cell body expression of ChR2-mCherry within the SNc and VTA. We excluded mice that exhibited ChR2-mCherry expression in either the IPN or the SUM (both regions known to express nNOS; (Rodrigo et al., 1994; Gonzalez-Hernandez and Rodriguez, 2000). The extent of cell body expression fell into three groupings (Fig. 3*A*; Table 3): group 1 exhibited robust ChR2-mCherry cell body expression in the PBP, SNc, VTAR, and RLi; group 2 exhibited robust ChR2-mCherry cell body expression in the PBP, SNc, and a dorso-lateral boundary region of the VTAR (where we did not see cell bodies in group 1); group 3 exhibited robust ChR2-mCherry cell body expression only in the PBP and SNc. In all cases, cell bodies exhibited robust expression of ChR2-mCherry, which was also often seen in long dendritic processes. Importantly, cell body and dendritic morphology appeared normal in neurons expressing mCherry (Fig. 3*B*), when compared to previous reports for GABA neurons in the VTA (Chieng et al., 2011; Margolis et al., 2012).

**Table 3.** ChR2-mCherry expression in cell bodies and axon terminals following VTA/SNc AAV injections in NOS1Cre-/+ mice

	Group 1				Group 2			Group 3		
Injection number	7	8	9	10	13	14	15	16	17	18
Cell bodies										
VTAR	+	++	++	+++	+++	+++	+++		+	+
RLi	+++	+++	+++	+++						
PBP	+++	+++	+++	+++	+++	+++	+++	++	+++	++
SNc	+++	+++	+++	+++	+++	+++	+++	++	+++	+
Axonal projections										
Septum										
MS	++	++	++	++						
HDB	+	+	+	+						
LS	+	+	+	+						
ST	+	+	+	+						
Striatum										
NAc (shell)	+	+	+	+						
VP	+++	+++	+++	+++						
NAc (core)				+						
Hypothalamus										
LH	+++	+++	+++	+++	+	+	+			
DM	+	+	+	+			+			
PO	++	++	++	++						
ZI	+	+	+	+						
Thalamus										
LHb	+	+	+	+						
MD				+						
VM	+	+	+	+						
Amygdala										
EAM				+						
AA				+						
Midbrain										
Mammillary	+									
Pons/medulla										
DR	+	+	+	++						
PMnR	+	+	+	++						
MnR	++	++	+	++						
PAG	+	+	+	+						
CLi	+	+	+	+						
NRO	+	+	+	+						

ChR2-mCherry expression density: +, very sparse expression; ++, modest expression; +++, dense expression. AA, amygdaloid area; CLi, caudal linear nucleus; DM, dorsomedial hypothalamic nucleus; DR, dorsal raphe nucleus; EAM, extended amygdala, medial part; HDB, horizontal limb of the diagonal band of Broca; LH, lateral hypothalamus; LHb, lateral habenula; LS, lateral septum; MD, dorsomedial nucleus of the hypothalamus; Mm, mammillary bodies; MnR, median raphe nucleus; MS, medial septum; NAc, nucleus accumbens; NRO, nucleus raphe obscurus PAG, periaqueductal gray; PBP, parabrachial pigmented nucleus; PMnR, paramedian raphe nucleus; PO, preoptic area; RLi, rostral linear nucleus; SNr, substantia nigra pars reticulata; ST, stria terminalis; VM, ventromedial thalamus; VP, ventral pallidum; VTAR, rostral ventral tegmental area; ZI, zona inserta. ^a^, cell bodies were restricted to the dorso-lateral boundary region of the VTAR and this was not seen in group 1 or group 3.

### When ChR2-mCherry expression was restricted to cell bodies in the PBP part of the VTA and the SNc, no axonal projections were found outside of the VTA and SNc

**Figure 4.**
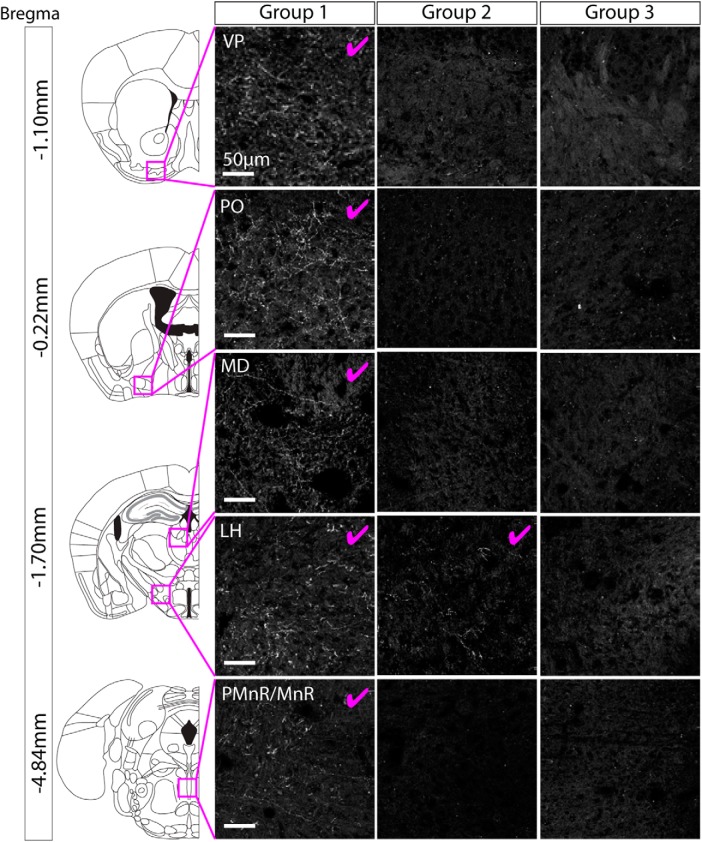
When ChR2-mCherry expression was restricted to cell bodies in the PBP part of the VTA and the SNc, no axonal projections were found outside of the VTA and SNc. Representative images of axon-expressed ChR2-mCherry for each group. group 1 exhibited extensive projections (for full summary, see Table 3) to multiple regions. Images are shown for the VP, PO, MD, LH, IPN, and PMnR/MnR, where the most extensive axonal expression was observed (pink tick indicates robust axonal expression). Group 2 exhibited sparse projections that were limited to the LH. Group 3 (which had cell body labeling restricted to the PBP and SNc) did not exhibit any axonal expression outside of the VTA and SNc.

For each mouse we conducted a full survey of the entire brain looking for ChR2-mCherry positive axonal projections. In brains from group 1 (which exhibited cell body labeling in the PBP, SNc, VTAR, and RLi), we observed extensive axonal projections in multiple regions (Fig. 4; Table 3), all shown previously to receive input from GABA and glutamate neurons in the VTA (Taylor et al., 2014). These projections were most dense in the ventral pallidum (VP), lateral hypothalamus (LH), and median raphe (MnR). In brains from group 2 (which exhibited cell body labeling in the PBP, SNc, and dorso-lateral part of the VTAR) we only reliably observed very sparse processes in the LH (Fig. 4; Table 3). In brains from group 3 (which exhibited robust cell body labeling only in the PBP and SNc), we did not observe any axonal projections outside of the VTA and SNc (Fig. 4; Table 3). On the basis of these expression patterns we can, therefore, draw two main conclusions. First, NOS1Cre+ neurons in the PBP and SNc do not send axonal projections outside of the VTA and SNC. Second, NOS1Cre+ neurons in the VTAR and RLi send extensive projections to multiple regions, including the VP, LH and MnR. All of these regions are known to receive input from the RLi (Del-Fava et al., 2007). It should be noted that in the case of group 2, where some sparse fibers were observed the LH, the cell body labeling in these cases was restricted to the dorso-lateral part of the VTAR only. In contrast in group 1 cell body labeling was observed throughout the VTAR.

### Cell body expression of ChR2-mCherry was colocalized with nNOS immunolabelling in the VTA, but in the SNc some neurons were TH+

**Figure 5.**
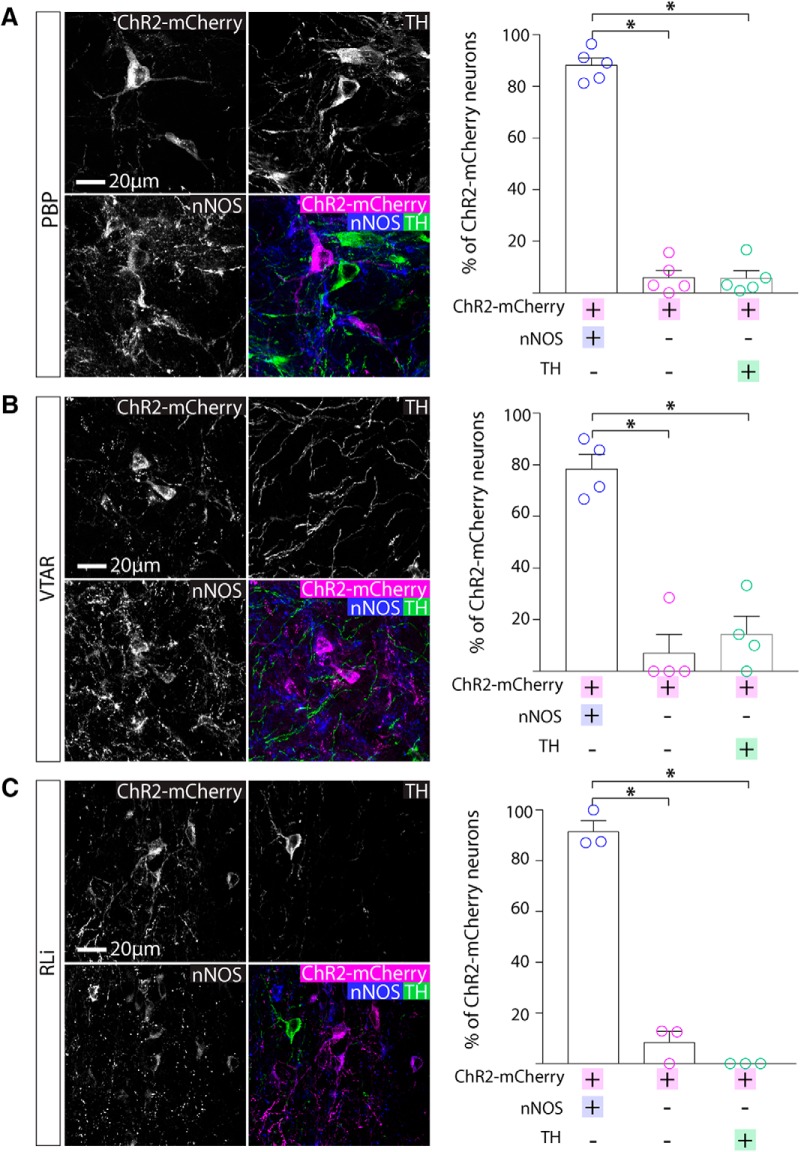
Cell body expression of ChR2-mCherry was colocalized with nNOS immunolabelling in the VTA. ***A***, Representative images of triple immunolabelling for ChR2-mCherry, nNOS, TH in the PBP. Graph shows the mean (±SEM) and individual data points for percentage of ChR2-mCherry that were colocalized with nNOS and/or TH (230 ChR2-mCherry+ cells, five mice). Almost all ChR2-mCherry+ neurons were nNOS+ and TH-. ***B***, Representative images of triple immunolabelling for ChR2-mCherry, nNOS, TH in the VTAR. Graph shows the mean (±SEM) and individual data points for percentage of ChR2-mCherry that were colocalized with nNOS and/or TH (40 ChR2-mCherry+ cells, four mice). Almost all ChR2-mCherry+ neurons were nNOS+ and TH-. ***C***, Representative images of triple immunolabelling for ChR2-mCherry, nNOS, TH in the RLi part of the VTA. Graph shows the mean (±SEM) and individual data points for percentage of ChR2-mCherry that were colocalized with nNOS and/or TH (155 ChR2-mCherry+ cells, three mice). Almost all ChR2-mCherry+ neurons were nNOS+ and TH-; *p < 0.05.

We next examined the degree of colocalization between ChR2-mCherry, nNOS, and TH in cell bodies in the PBP, SNc, VTAR, and RLi (*n* = 3–5 mice, 554 neurons). We conducted immunolabelling for nNOS and TH and examined colocalization with ChR2-mCherry. In the PBP (*n* = 5 mice; ANOVA: *F*_(2,12)_ = 290.0, *p* < 0.0001), VTAR (*n* = 4 mice; ANOVA: *F*_(2,9)_ = 35.27, *p* < 0.0001), and RLi (*n* = 3 mice; ANOVA: *F*_(2,6)_ = 213.9, *p* < 0.0001) nucleus, almost all ChR2-mCherry+ cells were nNOS+ and TH- (Fig. 5*A–C*).

**Figure 6.**
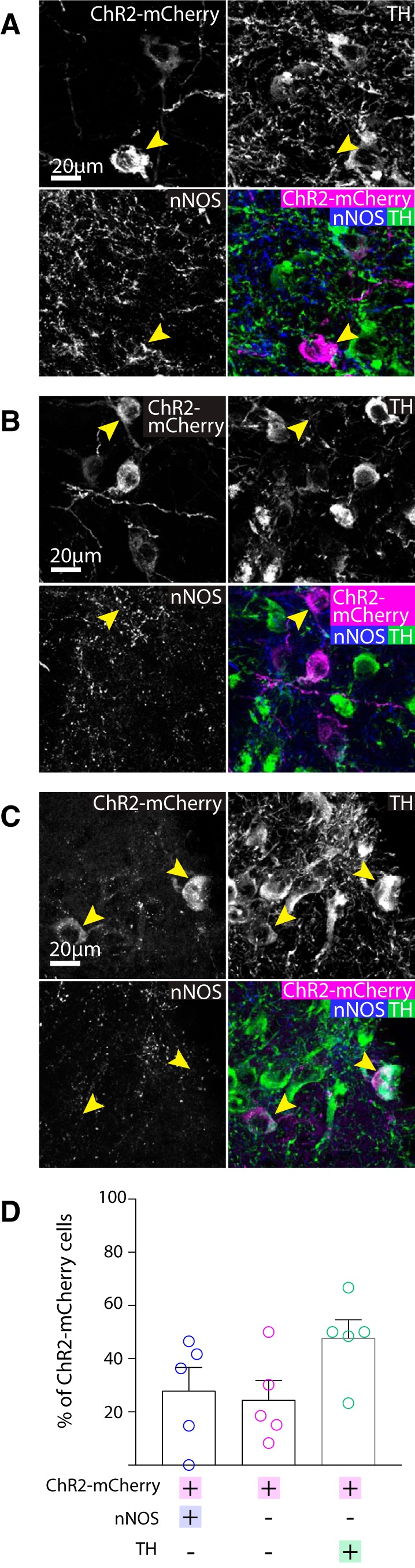
Cell body expression of ChR2-mCherry was mostly colocalised with either nNOS or TH in the SNc. Representative images of triple immunolabelling for ChR2-mCherry, nNOS, TH in the SNc, showing exemplar cells exhibiting either: ***A***, colocalisation of nNOS and ChR2-mCherry, but not TH. ***B***, expression of ChR2-mCherry, but neither nNOS nor TH. or ***C***, co-localisation of ChR2-mCherry and TH, but not nNOS. ***D***, Graph shows the mean (+SEM) and individual data points for percentage of ChR2-mCherry that were colocalised with nNOS and/or TH (129 ChR2-mCherry+ cells, five mice). Most ChR2-mCherry+ neurons were either nNOS+ or TH+. Yellow arrows indicate exemplar neurons.

**Figure 7.**
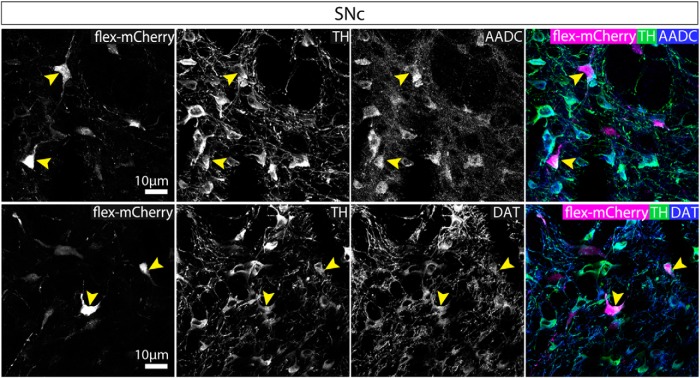
mCherry-expressing neurons that were also TH+, co-expressed AADC and DAT. Representative images of triple immunolabelling for mCherry, TH and AADC or mCherry, TH, and DAT. mCherry+ neurons that were TH+ were also AADC+ and DAT+. Yellow arrows indicate exemplar cell bodies, exhibiting triple colocalization.

In contrast, in the SNc similar numbers of neurons were ChR2-mCherry+ and/or nNOS+ and/or TH+ (ANOVA: *F*_(2,12)_ = 2.627, *p* = 0.1132). Although a majority of the ChR2-mCherry+ cells were nNOS+ (Fig. 6), surprisingly, around half of the ChR2-mCherry+ neurons in the SNc were TH+ (and nNOS-; Fig. 6). As observed in both the wild-type and VGATCre:RiboTag mice, nNOS antibody immunolabelling did not colocalize with TH in the SNc. It is possible, however, that these neurons appear immuno-negative for nNOS because they are either expressing very low levels of the enzyme (so that it is not detectable with the nNOS antibody), or that nNOS mRNA is being transcribed but the protein is not being synthesized currently. Because this result was somewhat unexpected, we wanted to replicate it with a different AAV. In this case, we injected AAV-hsyn-flex-mCherry into the SNc and lateral VTA. In cases where cell body labeling was restricted to neurons in the SNc and lateral VTA (*n* = 2), we again observed mCherry+ neurons that were also TH+, and we could not detect any axonal projections outside of the SNc and VTA. Furthermore, we also found that in all cases examined these TH+ neurons co-expressed aromatic L-amino acid decarboxylase (AADC) and the dopamine transporter (DAT), suggesting that they might be dopamine releasing (Fig. 7).

### Axonal expression of ChR2-mCherry+ was colocalized with GABAergic synaptic boutons in the VTA and SNc

**Figure 8.**
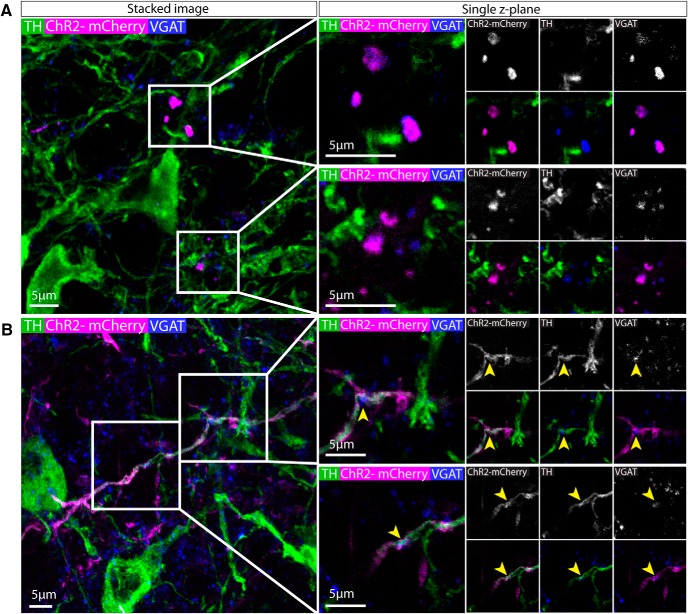
Axonal expression of ChR2-mCherry+ was colocalized with GABAergic synaptic boutons in the VTA and SNc. ***A***, Representative images of immunolabelling for ChR2-mCherry, VGAT, and TH in the PBP. ChR2-mCherry colocalizes with VGAT puncta in a single *z*-plane image suggesting the presence of GABAergic synapses. ***B***, Representative images of immunolabelling for ChR2-mCherry, VGAT, and TH in the SNc. ChR2-mCherry colocalizes with VGAT puncta in a single z-plane image suggesting the presence of GABAergic synapses. These puncta are also often TH+. Yellow arrows indicate exemplar puncta.

Taken together, our findings suggest that nNOS+ neurons in the PBP and SNc are GABAergic and do not project outside the VTA and SNc. To further examine their neurochemical identity, we examined single *z*-plane images of tissue immunolabelled for VGAT and TH. In the VTA and SNc, although VGAT antibodies do not resolve cell bodies well (as discussed earlier) they can reliably label processes, include putative presynaptic boutons. In the VTA, we commonly observed VGAT+ puncta colocalized with ChR2-mCherry and in close proximity to, but not colocalizing with, TH+ processes (Fig. 8*A*). This is consistent with the possibility that nNOS+ interneurons form inhibitory synapses onto dopamine neurons. In addition, in the SNc, we were able to locate some ChR2-mCherry+ fibers that were also colocalized with VGAT+/TH+ puncta (Fig. 8*B*).

### Axonal expression of ChR2-mCherry was colocalized with glutamatergic synaptic boutons in the VP and MnR

**Figure 9.**
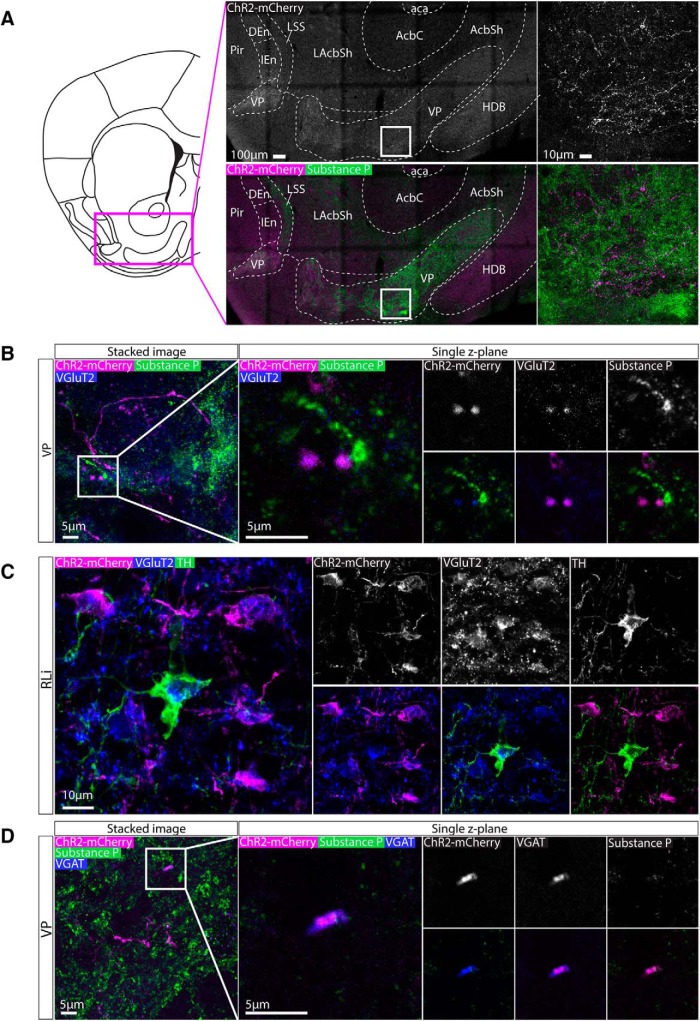
Axonal expression of ChR2-mCherry was colocalized with glutamatergic synaptic boutons in the VP. ***A***, Representative images of immunolabelling for ChR2-mCherry and substance P (which is highly expressed in the VP). Extensive innervation was observed in the VP compared to the neighboring parts of the NAc and septum. ***B***, High-magnification representative images of immunolabelling for ChR2-mCherry, substance P, and VGluT2 in the VP. Colocalization between ChR2-mCherry andVGluT2 puncta can be seen in single *z*-plane images, suggesting that these projections are glutamatergic. ***C***, Representative images of immunolabelling for ChR2-mCherry, VGluT2, and TH, in the RLi, occasionally also revealed cell bodies that expressed VGluT2. ***D***, High-magnification representative images of immunolabelling for ChR2-mCherry, substance P, and VGAT in the VP. On some occasions, colocalization between ChR2-mCherry and VGAT puncta was observed in single *z*-plane images, suggesting that some these projections are also be GABAergic.

The VP is the area that received the most prominent input from the nNOS+ neurons in the RLi nucleus, consistent with non-cell type-specific anterograde tracing approaches (Del-Fava et al., 2007). To examine this innervation in more detail, VP containing sections were immunolabelled for substance P (which delineates the VP) and either VGluT2 or VGAT. It can be clearly seen that ChR2-mCherry+ fibers were more prevalent in the VP (substance P+ region) compared to the horizontal limb of the diagonal band of Broca (HDB) and shell of the NAc (areas that receive sparse innervation; Fig. 9*A*). This innervation is present throughout the extent of the VP. ChR2-mCherry+ puncta could be clearly visualised among substance P+ puncta, and were commonly colocalized with VGluT2+ puncta (Fig. 9*B*). This is consistent with our observation that these projections originate mostly from cell bodies in the RLi and VTAR that are VGAT-/TH- and therefore putatively glutamatergic. Indeed, when we examined VGluT2 and ChR2-mCherry colocalization in the RLi, we observed some VGluT2+ cell bodies (as for GABAergic markers, it can be difficult to resolve cell bodies with antibodies for markers of glutamatergic neurons in the VTA) that were ChR2-mCherry+, consistent with our hypothesis that this is a predominantly glutamatergic population (Fig. 9*C*). Lastly, when we conducted immunolabelling for VGAT, we occasionally observed colocalization with ChR2-mCherry+ puncta, but these were less common than for VGluT2 (Fig. 9*D*).

**Figure 10.**
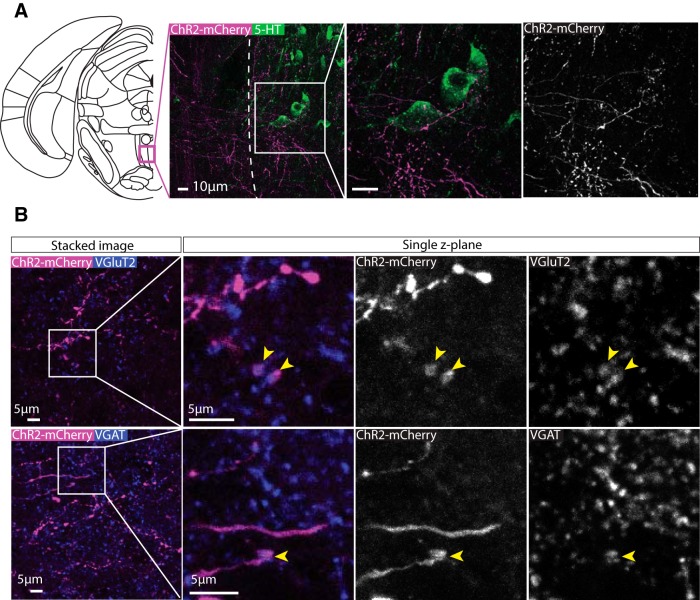
Axonal expression of ChR2-mCherry was colocalized with glutamatergic synaptic boutons in the MnR. ***A***, Representative images of immunolabelling for ChR2-mCherry and 5-HT (which is highly expressed in the MnR compared to nearby regions). Extensive innervation was observed in the MnR with axons often passing in close apposition to 5-HT[1] neurons. ***B***, Upper panels show high-magnification representative images of immunolabelling for ChR2-mCherry and VGluT2 in the MnR. Colocalization between ChR2-mCherry and VGluT2 puncta can be seen in single z-plane images, suggesting that these projections are glutamatergic. Lower panels show high-magnification representative images of immunolabelling for ChR2-mCherry and VGAT puncta in the MnR. On some occasions, colocalization between ChR2-mCherry and VGAT was observed in single z-plane images, suggesting that some these projections may also be GABAergic.

A second region that received extensive input was the MnR. Immunolabelling for serotonin (5-HT) revealed ChR2-mCherry+ terminals often in close proximity 5-HT+ neurons. (Fig. 10*A*). Similar to the VP, VGluT2+ (Fig. 10*B*) and VGAT+ (Fig. 10*C*) puncta colocalized with ChR2-mCherry+ puncta in single *z*-plane images.

## Discussion

Previous investigations of nNOS expression in the VTA and SNc have produced discrepant results with respect to the extent of its expression, which sub-regions within the VTA and SNc it is expressed in, and the degree of co-expression by dopamine neurons (Vincent and Kimura, 1992; Rodrigo et al., 1994; Gonzalez-Hernandez and Rodriguez, 2000; Klejbor et al., 2004; Gotti et al., 2005; Cavalcanti-Kwiatkoski et al., 2010; Mitkovski et al., 2012). We hypothesized that this variation in the literature was in part due to the use of different antibodies not validated specifically for the VTA and SNc. Consistent with this, we found immunolabelling absent in control tissue from nNOS-deficient mice. This highlights the importance of validating antibodies. Using a validated antibody, we show that nNOS+ neurons are present in the SNc, VTAR, PBP, and RLi, but not other parts of the VTA, including the PN. In addition, we show that nNOS+ neurons in the SNc and PBP are largely GABAergic, whereas those located in the RLi and VTAR are largely glutamatergic. These GABAergic neurons appear to be interneurons: despite the high levels of expression of an anterograde tracer in their cell bodies, we could not detect any axonal projections outside of the VTA and SNc. We also observed at the light microscope level these neurons making, what appeared to be, local GABAergic synaptic boutons, but this would need to be confirmed anatomically and functionally using electron microscopy and electrophysiology respectively. Across these regions, nNOS+ neurons make up <10% of the total GABA neuron population. In contrast, we found that nNOS+/glutamatergic neurons sent extensive projections to several regions, including the VP, LH and MnR.

Previously, it has been demonstrated that GABA neurons in the VTA make anatomically-defined local synaptic connections with dopamine and non-dopamine neurons in the VTA (Omelchenko and Sesack, 2009). Moreover, functional optogenetic stimulation of VTA GABA neurons can evoke fast GABAA-receptor-mediated synaptic currents in dopamine neurons in the VTA (Tan et al., 2012; van Zessen et al., 2012). Activation of this local GABAergic microcircuit can generate a conditioned place aversion and reduce food consumption (Tan et al., 2012; van Zessen et al., 2012). It was not clear, however, whether the GABA neurons that made these local synaptic connections were also the same GABA neurons that send long-range projections to other regions such as the striatum (Brown et al., 2012; Taylor et al., 2014). Our findings suggest that at least one subset of these neurons are local GABAergic interneurons. Moreover, because these neurons have a distinct molecular identity (i.e., nNOS expression), they are experimentally tractable (e.g., by using cell type-specific functional and anatomic techniques in NOS1Cre mice). This approach could be further refined using intersectional genetics (e.g., to limit expression-based GABAergic or glutamatergic identity). A number of technical considerations must be taken into account with respect to this conclusion. Firstly, it may be that their axons did not readily transport the fluorescent markers that we used and/or the expression of those markers caused some axonal damage to the neurons. Secondly, although we were unable to detect any axonal projections of these neurons outside of the VTA and SNc, it remains possible that they send some sparse projections which we overlooked, despite very careful inspection of whole brains. There are several reasons why we consider these possibilities to be unlikely. First, we carefully examined neurons for overall health and they appeared normal. Moreover, we have previously used the same AAV to label dopamine neurons in the VTA and hypothalamic neurons without any detectable effects on morphology, physiology, or behavior (Viskaitis et al., 2017; Sandhu et al., 2018). Second, nNOS+ neurons in the VTAR and RLi did exhibit extensive axonal projections, suggesting that mCherry/ChR2 can be visualised in the axons of a neighboring (in parts anatomically overlapping) population. Moreover, these neurons exhibited similar levels of cell body and dendritic labeling when compared to nNOS+ neurons in the PBP and SNc. Taken together, it would therefore be surprising if axonal transport of mCherry/ChR2 was completely absent in one of these populations but not the other.

One intriguing observation was that a subset of nNOS-Cre+ neurons in the SNc were TH+. Importantly, these neurons do not send projections outside the SNc. It is, of course, a canonical view of the mesocorticolimbic dopamine system that TH+ neurons in the SNc send extremely dense axonal projections to several target regions, most notably the striatum (Matsuda et al., 2009). Not withstanding the caveats discussed in the previous paragraph, our findings suggest, however, that a subset of TH+ neurons in the SNc are local interneurons (or at least have dramatically more limited axonal projections than typical SNc dopamine neurons). Interestingly, there is evidence for TH+ GABAergic interneurons in the striatum (Dubach et al., 1987; Tashiro et al., 1989; Meredith et al., 1999; O'Byrne et al., 2000; Mao et al., 2001; Petroske et al., 2001; Ibáñez-Sandoval et al., 2010; Unal et al., 2011; Ünal et al., 2015; Xenias et al., 2015). Optogenetic stimulation of these TH+ neurons in the striatum fails to elicit any detectable release of dopamine (Ibáñez-Sandoval et al., 2010; Xenias et al., 2015). In addition, they do not express AADC, dopamine, or DAT (Xenias et al., 2015). Instead, optogentic activation of these neurons elicited GABA-mediated IPSCs in mediam spiny neurons (Ibáñez-Sandoval et al., 2010; Xenias et al., 2015). Colocalization with GABA synthesizing enzymes GAD65 and GAD67 has also been reported (Betarbet et al., 1997; Cossette et al., 2005; Mazloom and Smith, 2006; Tandé et al., 2006; San Sebastián et al., 2007). Notably, this interneuron population is considered to be distinct from the nNOS+ interneurons in the striatum (Ibáñez-Sandoval et al., 2010; Tepper et al., 2010). In contrast, we observed co-expression of AADC and DAT in our subset of non-projecting TH+ neurons in the SNc, suggesting that they may be dopaminergic. It will be important, therefore, to establish whether they release dopamine. Our examination of synaptic terminals in the SNc suggest that some at least may be GABAergic. It is also not clear whether these TH+ interneurons would be mistaken for TH+, long-range projecting dopamine neurons in studies where TH-GFP or TH-Cre mice are used to identify and/or manipulate dopamine neurons.

Glutamate neurons are found sparsely distributed throughout the SNc and VTA, although at a greater density in more medial regions of the VTA (Yamaguchi et al., 2007, 2011, 2013, 2015; Nair-Roberts et al., 2008; Morales and Root, 2014; Root et al., 2016; Morales and Margolis, 2017). Some of these neurons co-release dopamine or GABA (Stuber et al., 2010; Tecuapetla et al., 2010; Root et al., 2014b; Zhang et al., 2015; Yoo et al., 2016). They make local synaptic connections with dopamine and non-dopamine neurons and send projections to several regions including the striatum (Dobi et al., 2010; Hnasko et al., 2012; Root et al., 2014a,b; Taylor et al., 2014). Interestingly, optogenetic excitation of VTA glutamate neurons can have rewarding and aversive effects, depending in part on the site of stimulation, suggesting some functional heterogeneity (Root et al., 2014a; Wang et al., 2015; Qi et al., 2016; Yoo et al., 2016). We have found the nNOS is expressed by glutamate neurons in the VTAR and RLi that send projections most densely to the VP, LH, and MnR. This is consistent with previous reports of non-cell type-specific anterograde labeling of projections from the RLi to the VP, but not NAc (Del-Fava et al., 2007). Based on reports of the full projectome of glutamate neurons in the VTA, which includes extensive projections to regions such as the NAc (Hnasko et al., 2012; Taylor et al., 2014; Qi et al., 2016), we conclude that nNOS+ neurons represent a projection-specific sub-group of this population. As is the case for nNOS+ GABA neurons in the PBP and SNc, because nNOS+ glutamate neurons have a distinct molecular identity understanding their function will be experimentally tractable.

In conclusion, our findings indicate that nNOS is expressed by neurochemically- and anatomically-distinct neuronal sub-groups in a sub-region-specific manner within the VTA and SNc.

## Synthesis

Reviewing Editor: Christian Lüscher, University of Geneva Decisions are customarily a result of the Reviewing Editor and the peer reviewers coming together and discussing their recommendations until a consensus is reached. When revisions are invited, a fact-based synthesis statement explaining their decision and outlining what is needed to prepare a revision will be listed below. Note: If this manuscript was transferred from JNeurosci and a decision was made to accept the manuscript without peer review, a brief statement to this effect will instead be what is listed below.

While it would greatly enhance the appeal of the paper to show connectivity with electrophysiology, I understand the authors argument that this would delay the publication of the anatomical findings. Given the now much balanced discussion, I believe we can go forward with the publication.
